# MUC13 contributes to rewiring of glucose metabolism in pancreatic cancer

**DOI:** 10.1038/s41389-018-0031-0

**Published:** 2018-02-22

**Authors:** Sonam Kumari, Sheema Khan, Subash C. Gupta, Vivek K. Kashyap, Murali M. Yallapu, Subhash C. Chauhan, Meena Jaggi

**Affiliations:** 1 Department of Pharmaceutical Sciences and Center for Cancer Research, University of Tennessee Health Science Center, Memphis, TN USA; 2 Department of Biochemistry, Institute of Science, Banaras Hindu University, Varanasi, India

## Abstract

Pancreatic tumors are rewired for high-glucose metabolism and typically present with exceptionally poor prognosis. Recently, we have shown that MUC13, which is highly expressed in pancreatic tumors, promotes tumor progression via modulation of HER2 receptor tyrosine kinase activity. Herein, we investigate a novel, MUC13-mediated molecular mechanism responsible for higher glucose metabolism in pancreatic tumors. Our results demonstrate that MUC13 expression leads to the activation/nuclear translocation of NF-κB p65 and phosphorylation of IκB, which in turn upregulates the expression of important proteins (Glut-1, c-Myc, and Bcl-2) that are involved in glucose metabolism. MUC13 functionally interacts and stabilizes Glut-1 to instigate downstream events responsible for higher glucose uptake in pancreatic cancer cells. Altered MUC13 expression by overexpression and knockdown techniques effectively modulated glucose uptake, lactate secretion, and metastatic phenotypes in pancreatic cancer cells. NF-κB inhibitor, Sulfasalazine, abrogates the MUC13 and Glut-1 interaction, and attenuates events associated with MUC13-induced glucose metabolism. Pancreatic ductal adenocarcinoma (PDAC) patient tissue samples also show a positive correlation between the expression of these two proteins. These results delineate how MUC13 rewire aberrant glucose metabolism to enhance aggressiveness of pancreatic cancer and revealed a novel mechanism to develop newer therapeutic strategies for this exceptionally difficult cancer.

## Introduction

Pancreatic cancer (PanCa) will be the second leading cause of cancer-related deaths very soon as per future projections [1]. Due to the late diagnosis, chemo-resistance and metastasis, 5-year survival rate still remains <8% [1]. The pancreas has an anatomically inaccessible location that prevents routine examination, early stage diagnosis, and timely surgical resection of tumor(s). In addition, because of highly aggressive nature, pancreatic cancer cells disseminate rapidly leading to early metastasis of the disease. At this stage, available treatment options are only moderately successful to extend the patient’s survival rate. Therefore, it is highly important to understand and delineate the molecular pathways that lead to increased tumor aggressiveness and poor patient survival rate. One such unique characteristic physiology of pancreatic tumors includes a hostile and tumor microenvironment, which favors biochemical and metabolic adaptations to facilitate pancreatic tumor growth and metastasis. Alterations in metabolic pathways, which have a common feature of increased glucose uptake and its conversion to lactate, are highly responsible for cancer progression [2].

Cancer cells undergo an oncogene addiction, which makes them highly dependent on the activity of an oncogene for survival and proliferation [3]. The fibrotic pancreatic tumor microenvironment enables pancreatic cancer cells to rely on alternative sources of nutrients and adapt distinctive approaches to obtain them. Most cancer cells exhibit enhanced aerobic glycolysis, known as the Warburg effect, an alteration in glucose metabolism resulting in an acidic tumor microenvironment that facilitates local invasion of tumor cells [4]. Warburg effect is the enhanced conversion of glucose to lactate observed in tumor cells, even in the presence of normal levels of oxygen. Glucose is the major metabolic precursor that supports the Warburg effect and glucose transporter-1 (Glut-1) mediates cellular glucose transport required to fuel anaerobic metabolism in proliferating cancer cells [5]. The increased lactate production results in enhanced tumorigenic characteristics such as cell invasion, migration, metastasis of the tumor and correlates with tumor reoccurrence [6]. Thus, oncogenic networks required for this process, are highly desirable to develop novel strategies for selective targeting of aberrant pancreatic tumor metabolism and tumor microenvironment. Pancreatic cancer metabolism is dramatically rewired by oncogenic KRAS that induces a series of metabolic alterations, which includes enhanced glycolysis and glutaminolysis, resulting in enhanced cell growth and proliferation [7]. Hypoxia is one of the factors in the pancreatic tumors that leads to enhanced glucose metabolism, hypoxia-inducible factor 1 (HIF-1) being an important regulator of cellular oxygen homeostasis [8]. NF-κB plays an important role to regulate the metabolic adaptation in normal and cancer cells by controlling the balance between the utilization of glycolysis and mitochondrial respiration [9].

Mucins are a family of high molecular weight and glycosylated proteins that play significant roles in pancreatic cancer pathogenesis [10]. MUC13 protein, is a transmembrane mucin, which is aberrantly expressed in pancreatic cancer [11] and enhances pancreatic tumor progression through various mechanisms; one such mechanism is being mediated through its interaction with HER2, a member of EGFR family [12]. In the present study, we report a novel role of MUC13 in metabolic reprogramming of pancreatic cancer. We demonstrate the metabolic alterations induced by MUC13 in pancreatic cancer cells and the underlying molecular mechanisms that drive the associated tumorigenic characteristics. Our studies indicate that the MUC13-induced metabolic alterations require NF-κB activation that precedes the triggering of events in controlling glucose metabolism in pancreatic cancer cells. In addition, for the first time, we report a novel interaction between MUC13 and Glut-1 and a positive correlation of these proteins in pancreatic ductal adenocarcinoma (PDAC) patient’s tissue samples. Briefly, our study delineate the role of MUC13 in aberrant glucose metabolism in pancreatic cancer and suggest it as a novel molecular target for therapeutic intervention of this frightening disease.

## Results

### MUC13 enhances glucose metabolism and invasiveness in PDAC cells

**Fig. 1:**
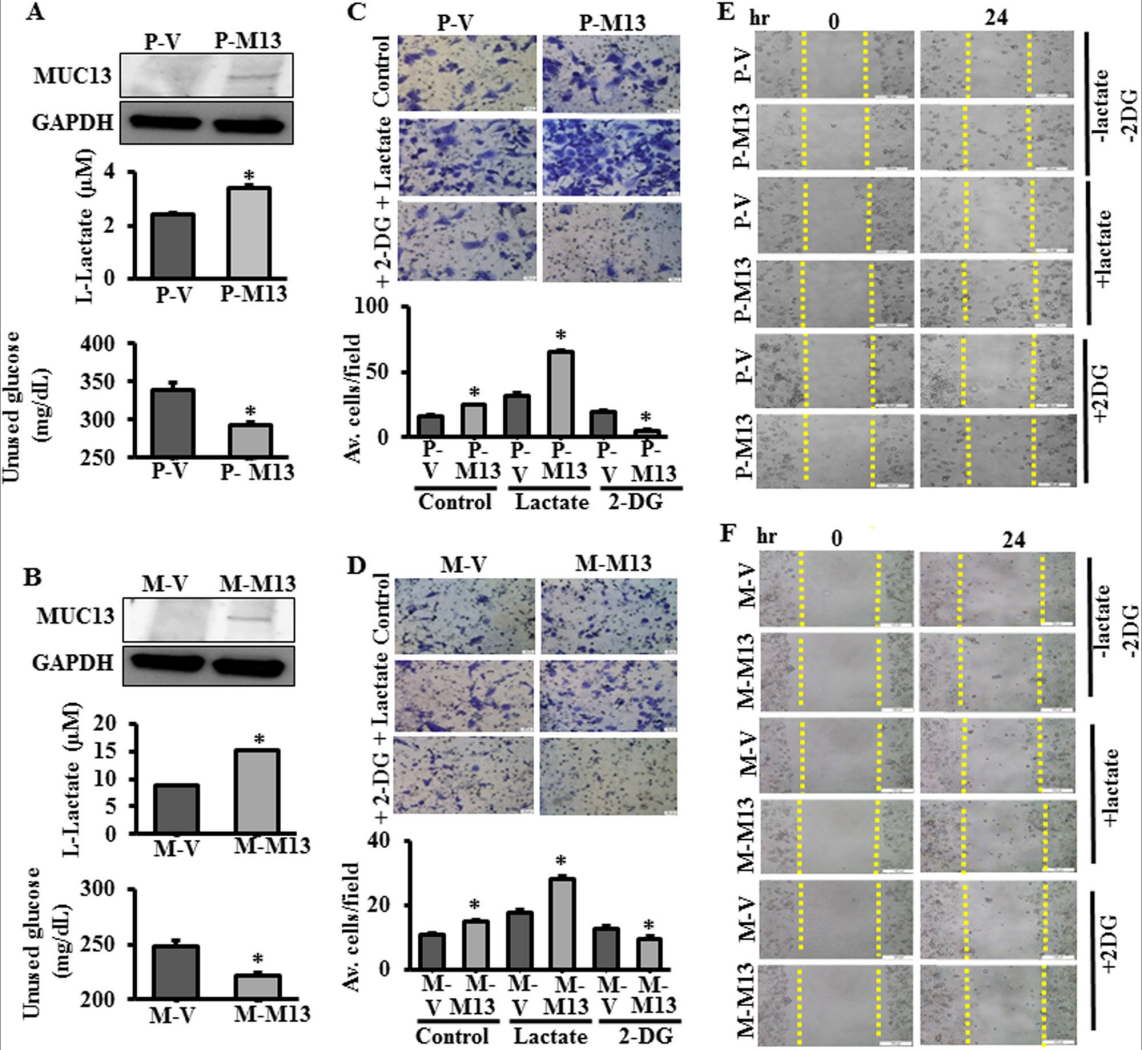
MUC13 enhances glucose metabolism in PDAC cells. Expression of MUC13 shown by western blotting, lactate and glucose assay in MUC13-null Panc-1 (P-V) and stably expressing MUC13 (P-M13) cells (**a**) and in MUC13-null MiaPaca (M-V) and stably expressing MUC13 (M-M13) cells (**b**). Invasion assay performed in P-V and P-M13 (**c**) and M-V and M13 cells (**d**) after treatment with lactate (2 mM) and 2-DG (10 mM). Images were captured after 24 h and the relative number of invasive cells was quantified. Wound-healing (scratch) assay performed in P-V and P-M13 (**e**) and M-V and M13 cells (**f**) after the treatment with lactate (2 mM) and 2-DG (10 mM). The images were captured at 0 h and 24 h. *n* = 3; *p < 0.05

Processes in malignant transformation involve enhanced glucose uptake and lactate production which consequently lead to defects in the expression of glycolytic enzymes and metabolite transporters and in oncogenic alterations [13]. In order to investigate the role of MUC13 on glycolytic properties of PanCa cells, glucose and lactate assays were performed in MUC13-null Panc-1/MiaPaca cells (P-V/M-V) or stably MUC13 overexpressing (P-M13/M-M13) cells. We observed significantly (*p* < 0.05) higher upregulation of l-lactate production and glucose consumption in MUC13-expressing cells as compared to MUC13-null control cells (Fig. 1a, b). Whereas, MUC13 knockdown in HPAF-II cells (sh-M13) demonstrated inhibition of lactate secretion and glucose consumption (Supplementary Figure S1A-C). Therefore, our results demonstrate that MUC13 enhances glucose metabolism of pancreatic cancer cells. Previous reports demonstrate that MUC13 enhances invasion and migration in pancreatic cancer cells [11]. In this study, our observations suggest that MUC13 expression modulates glucose consumption in PDAC cells, therefore, we sought to investigate whether MUC13 induced changes in proliferation, invasion, and migration are mediated through altered glucose metabolism. We demonstrate that MUC13 expression (P-M13 and M-M13) enhances cell proliferation (Supplementary Figure 1D), invasion (Fig. 1c, d), and migration (Fig. 1e, f) that can be further enhanced by adding lactate (2 mM), an end product of aerobic glycolysis, to the media in which cells are cultured. This effect, however, was abrogated by inducing glucoprivic conditions using 2-deoxyglucose (2-DG, 10 mM; Fig. 1c–f). These results suggest that MUC13 influenced metastatic characteristics of PDAC cells are partly the consequence of its modulation of the glucose metabolism.

### MUC13 upregulates c-Myc expression and the downstream effectors of glucose metabolism

**Fig. 2:**
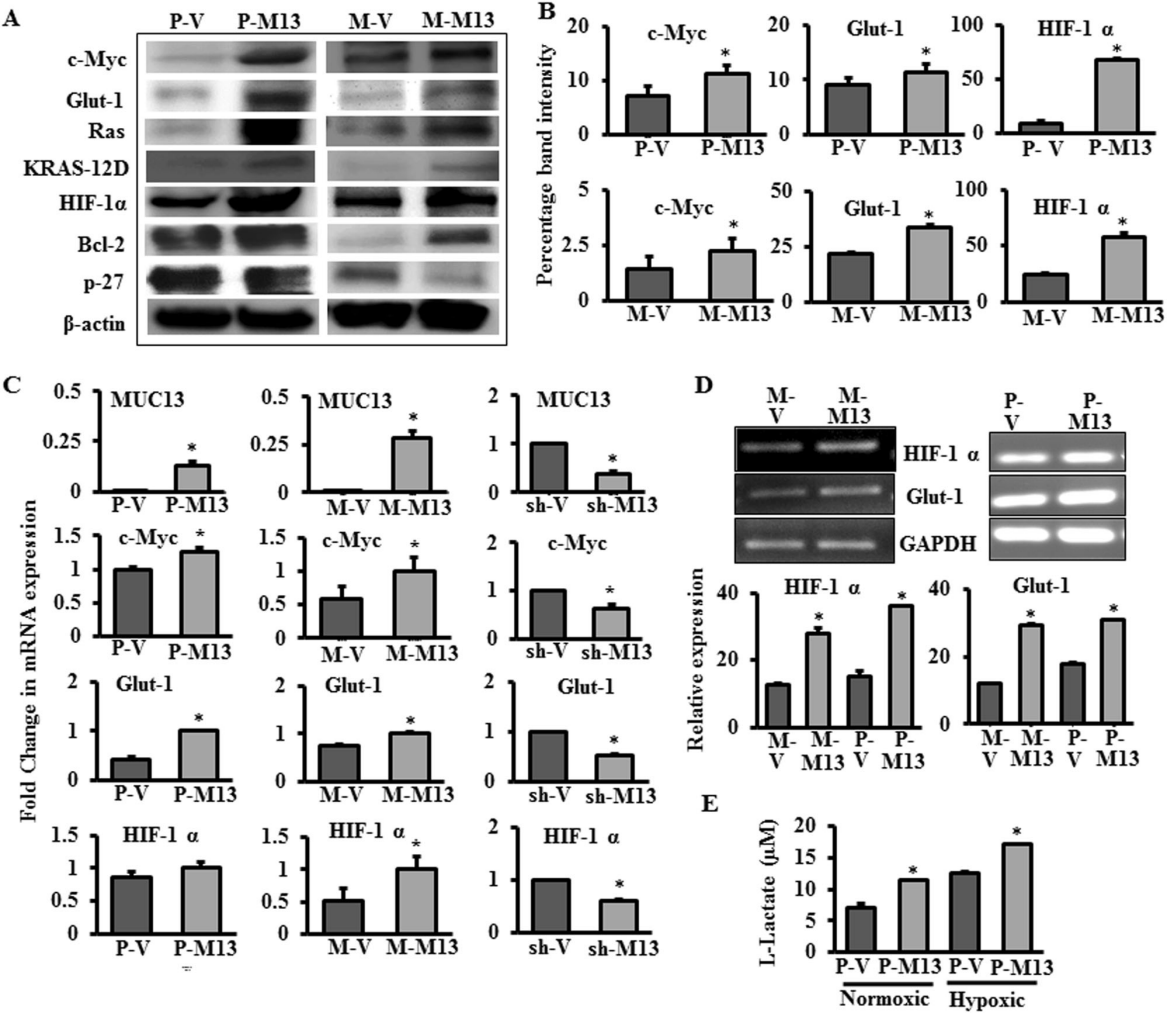
MUC13 regulates protein and mRNA expression of key factors involved in glucose metabolism. **a** Western blot showing the expression level of indicated proteins involved in glucose metabolism. Whole-cell lysates were collected from cells and the levels of protein expression were determined. **b** Densitometric analysis of bands for c-Myc, Glut-1 and HIF-1α. *n* = 2; *p < 0.05. **c** qPCR depicting changes in mRNA expression in MUC13-null (P-V/M-V) cells and MUC13-expressing (P-M13/M13) Panc-1 and MiaPaca cells; MUC13-expressing (sh-V) and knockdown (sh-M13) HPAF-II cells, *p < 0.05. **d** Semi-quantitative PCR performed to determine the expression of HIF-1 α and Glut-1 on MUC13 expression in PanCa cells and bars representing relative quantification of HIF-1α and Glut-1 expression. **f** Lactate assay performed in Panc-1 cells under normoxic and hypoxic conditions. *p < 0.05

Since our results showed a marked upregulation of glucose uptake and lactate production, we evaluated effects of MUC13 expression on the important proteins involved in enhanced cancer cell metabolism. Immunoblotting and immunofluorescence analysis demonstrated an enhanced expression of c-Myc (Fig. 2a, b) and its nuclear localization (Supplementary Figure S2A) in MUC13-expressing Panc-1 and MiaPaca cells (P-M13/M-M13). c-Myc is a master regulator of downstream processes that are involved in cell growth and metabolism [14]. The changes in c-Myc expression were accompanied by overexpression of Glut-1, which ensures accumulation of glucose in the cell and secretion of lactate out of the cell (Fig. 2a, b and Supplementary Figure S2B). In addition, increased expression of oncogenic KRAS was observed in MUC13-expressing cells that is known to maintain pancreatic tumors through regulation of anabolic glucose metabolism [7]. One of the mediators of KRAS-induced transcriptional changes of metabolic genes is known to be HIF-1α, and its expression was found to be significantly higher upon MUC13 expression (Fig. 2a, b and Supplementary Figure S3) [7]. The mean fluorescence intensities for c-Myc, Glut-1, and HIF-1α were also quantified using Zen software (Supplementary Figure S4A and B). In addition, MUC13-expressing cells were found to modulate proteins associated with cell death such as activation of anti-apoptotic protein, Bcl-2, and inhibition of tumor suppressor/cell cycle inhibitor, p27 (Fig. 2a). Immunoblotting and Immunofluorescence results were confirmed using quantitative PCR (qPCR) analysis (Fig. 2c) and semi-quantitative PCR (Fig. 2d) showing increased mRNA expression of c-Myc, Glut-1, and HIF-1α in MUC13-expressing Panc-1 and MiaPaca cells which was inhibited on MUC13 knockdown in HPAF-II cells (sh-MUC13). The activation of HIF-1α prompted us to investigate whether MUC13 typically demands hypoxic environment to contribute to adaptive mechanisms of enhanced cell metabolism. Our results showed that the induced hypoxic conditions accompany enhanced production of lactate in MUC13-expressing cells as compared with MUC13-null cells (Fig. 2e). These results indicate that MUC13 contributes to survival adaptation in hypoxic environment leading to enhanced metabolic changes for the survival of PanCa cells. These data suggest that MUC13 expression induce rewiring of oncogenic signaling pathways that contribute to adaptive cellular mechanisms in tumor microenvironment.

### MUC13 expression leads to the TNF-induced activation/nuclear shuttling of NF-κB p65

**Fig. 3:**
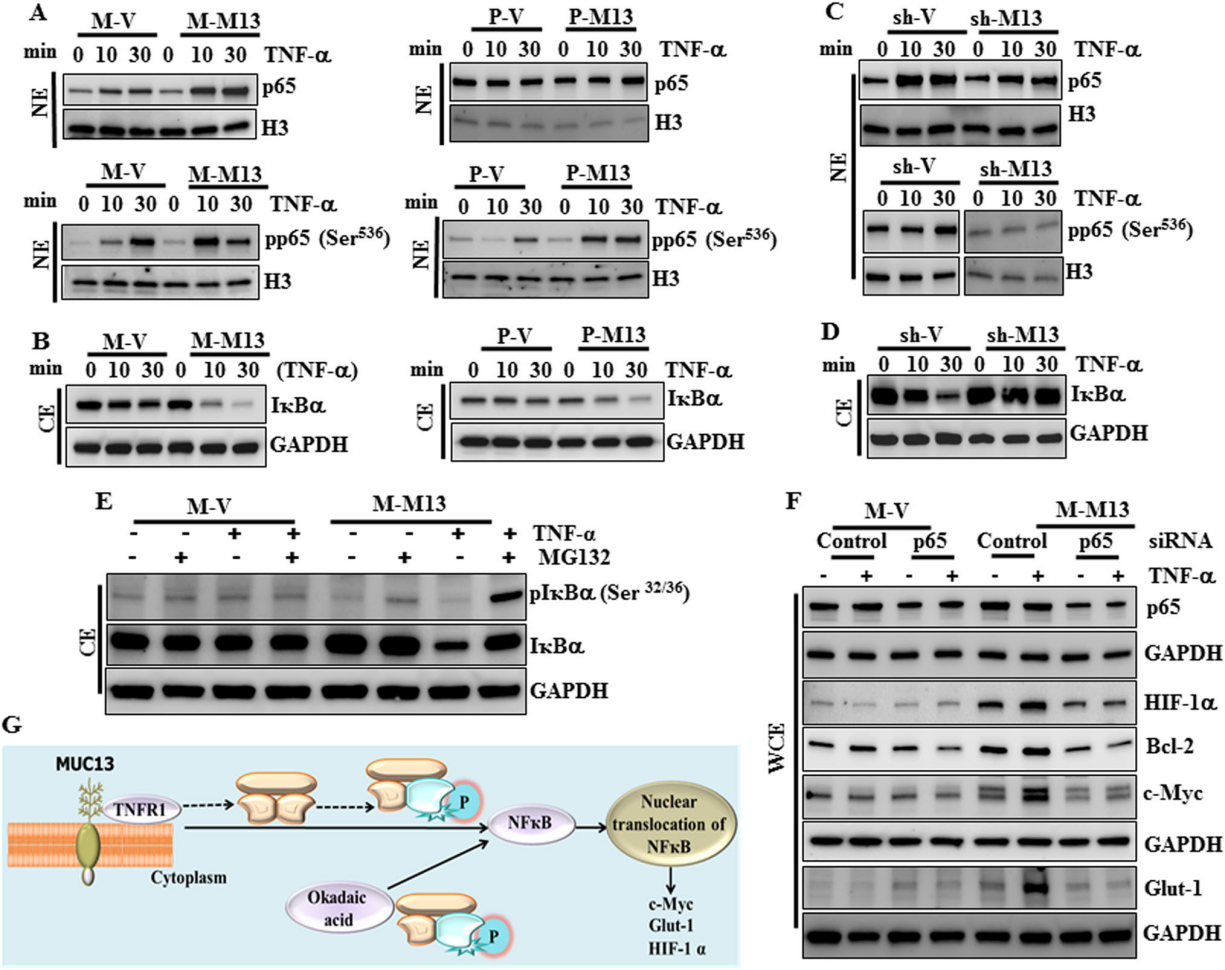
MUC13 expression leads to enhanced activation/nuclear translocation of NF-κB p65. **a** Western blots showing the expression level of p65 and phospho-p65 in M-V/M-M13 and P-V/P-M13 cells. Cells were spiked with TNF-α at a concentration of 20 ng/ml for 0, 10, and 30 min, and the nuclear extracts prepared. Histone H3 served as the internal control. **b** Western blots indicating changes in the expression of IκBα shown in the cytoplasmic extracts in aforementioned cell lines. **c**, **d** Western blots showing the expression level of p65, phospho-p65 and IκBα in the nuclear and cytoplasmic extracts of MUC13-expressing (sh-V) and MUC13 knockdown (sh-M13) HPAF-II cells. **e** Western blotting depicting the degradation of pIκB α and IκB α after treatment with MG132 (1 h, 10 µM) in presence of TNF-α stimulation (20 ng/ml, 30 min). **f** Western blotting depicting the effect of p65 silencing on the metabolism involved proteins in presence of TNF-α stimulation (5 ng/ml for 24 h) **g** Schematic representation depicting an activation of NFҡB on IҡB phosphorylation in presence of MUC13 following TNF-α or okadaic acid stimulation. This triggers NFҡB translocation to the nucleus and induction of target gene expression

We further wanted to investigate the signaling pathways that might be involved in the MUC13 influenced aberrant glucose metabolism in PanCa cells. Our results demonstrate that MUC13 expression facilitates tumor necrosis factor (TNF)-induced activation/phosphorylation of NF-κB p65 at Ser536 and its nuclear translocation in Panc-1 and MiaPaca (P-M13 and M-M13) cells. Although, TNF-α-induced p65 nuclear accumulation (0–30 min) was also observed in vector control cells (M-V and P-V), but a remarkable increase was observed upon MUC13 expression (M13 and P-M13), suggesting its involvement in the MUC13-mediated signaling mechanisms (Fig. 3a). These events were observed with concomitant degradation/decrease in the expression levels of IκBα (NF-κB repressor) in the cytoplasm, which is ultimately known to release its inhibition from NF-κB p65 (Fig. 3b) and accompany its translocation to the nucleus [15]. Similar results were corroborated on MUC13 knockdown in HPAF-II cells, which indicated decreased nuclear accumulation and phosphorylation of NF-κB p65 (Fig. 3c) as well as cytoplasmic IκBα degradation in MUC13 knockdown cells (sh-M13; Fig. 3d). These results suggest the association of MUC13 to the promotion of TNF-α-induced NF-κB activation. To investigate whether MUC13-induced activation of NF-κB is TNF dependent, we performed similar experiments following stimulation with Okadaic acid (OA) and examined the activation/phosphorylation status of NF-κB in MUC13-expressing cells. Our results indicated enhanced cytosolic IκBα degradation and nuclear NF-κB p65 phosphorylation upon OA treatment in MUC13-expressing (M-M13) cells (Supplementary Figure S5A and B). These results indicate that MUC13 induces NF-κB activation, which is independent of the mode of stimulation and may involve distinct mechanisms augmenting NF-κB activation. One such mechanism may be similar to the one discussed previously in colon cancer, which suggests the role of MUC13 in augmented recruitment of cIAP1 to the TNFR/RIPK1 complex, and promotion of RIPK1 ubiquitination by cIAP1, which ultimately leads to NF-κB activation and downstream NF-κB-regulated gene expression [16]. We then examined whether TNF-α-induced IκBα degradation is due to IκBα phosphorylation, and used a proteasome inhibitor, MG132 to block IκBα degradation. Western blotting analysis revealed TNF-α-induced phosphorylation/degradation of IκBα only in MUC13-expressing cells (M-M13) but not in vector control cells (M-V) (Fig. 3e), which was further restored on MG132 treatment. These results confirm the role of MUC13 in the induction of NF-κB activation via IκBα phosphorylation and degradation.

In order to determine whether NF-κB is required for MUC13-mediated alteration of glucose metabolism, we examined the expression of important proteins involved in regulation of glucose metabolism on silencing p65 in MUC13-null or stably expressing MiaPaca cells. Following TNF-α exposure for 24 h, MUC13-expressing cells exhibited increased expression of c-Myc, HIF-1α, Bcl-2, and Glut-1 as compared to MUC13-null cells. This increase, however, was repressed by silencing of p65 (Fig. 3f). These results suggest that NF-κB is required for MUC13-induced expression of proteins associated with enhanced glucose metabolism in PanCa cells (Fig. 3g).

### MUC13 interacts with Glut-1 and this interaction is disrupted by NF-κB inhibition

**Fig. 4:**
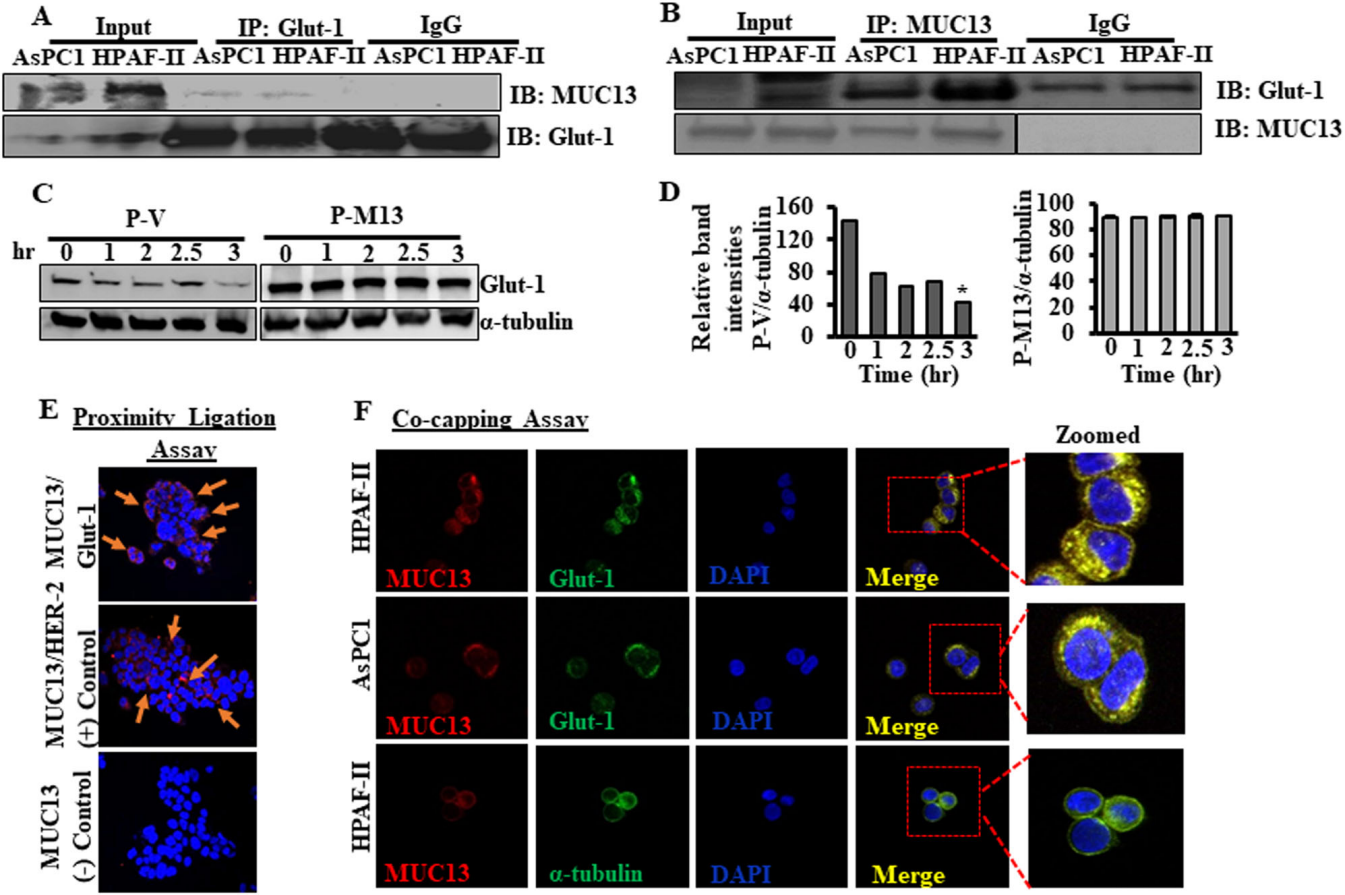
MUC13 interacts with Glut-1. **a**, **b** Reciprocal co-immunoprecipitation assay performed between MUC13 and Glut-1 using whole-cell protein lysates from MUC13-expressing, AsPC1, and HPAF-II cells. **c** Cycloheximide chase assay depicting the stability of Glut-1 in P-V and P-M13 cells. Cells were treated with cycloheximide (10 μM) at different indicated time points followed by lysate preparation and Western blotting. Alpha-tubulin (α-tubulin) served as an internal control. **d** Bars representing relative quantification of the blots. **e** In situ proximity ligation assay (PLA) performed using HPAF-II cells with the Duolink Red starter kit. DAPI was used as a counter stain for the nucleus. MUC13/Her2 served as positive control, and MUC13 alone as negative control. The co-localization between MUC13/Glut-1 and MUC13/Her2 has been indicated by arrows. **f** Co-capping assay to demonstrate interaction between MUC13 and Glut-1 in HPAF-II and AsPC1 cells. HPAF-II cells with anti-α tubulin is a negative control for this experiment. Images were captured at ×400

**Fig. 5:**
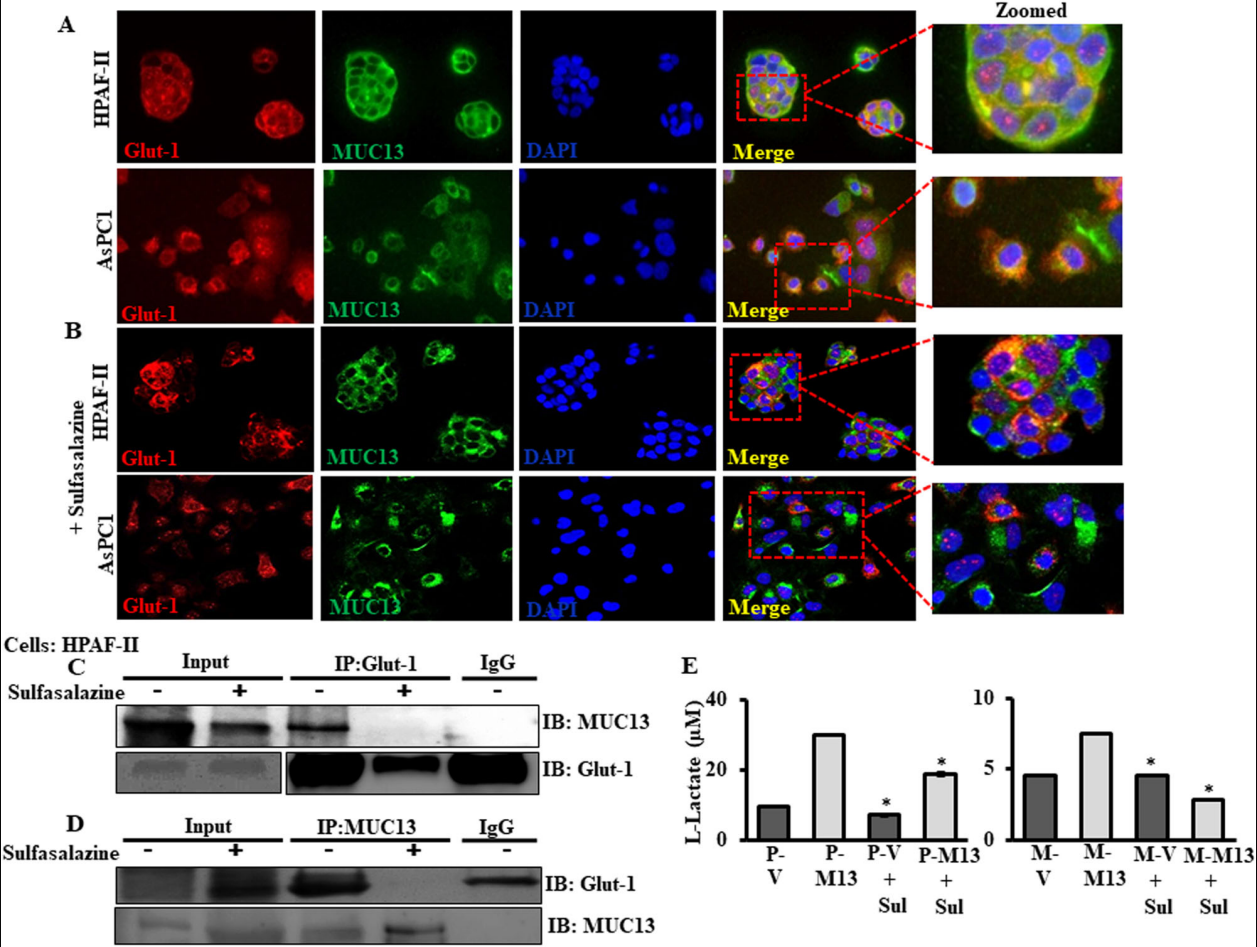
MUC13-Glut-1 interaction is disrupted by Sulfasalazine. **a** Confocal immunofluorescence of MUC13 and Glut-1 in MUC13-expressing, HPAF-II, and AsPC1 cells depicting co-localization (yellow color). Cells were cultured in 4-well chambered slides, fixed, permeabilized, stained with indicated antibodies and analyzed for confocal microscopy. DAPI was used as a counter stain for the nucleus. **b** Cells were treated with sulphasalazine (0.9 mM) for 24 h and processed for immunostaining and confocal microscopy. Images were captured at ×400. **c**, **d** Reciprocal co-immunoprecipitation assay between MUC13 and Glut-1 performed using protein lysates from HPAF-II cells. Cells were treated with sulfasalazine (0.9 mM) for 24 h and protein lysates prepared. The immunoprecipitates were resolved on a 10% gel, and followed by immunoblotting with anti-MUC13 and anti-Glut-1 antibodies. **e** Lactate assay was performed in MUC13-expressing and MUC13-null cells after treatment with or without sulfasalazine. Cell culture media was collected after 48 h to measure the amount of l-lactate concentration using lactate assay kit

Our results demonstrate increased Glut-1 expression in Panc-1 and MiaPaca cells while MUC13 is exogenously overexpressed (Fig. 2a), thus it is warranted to investigate molecular crosstalk between these proteins. Our investigations revealed a direct molecular interaction of MUC13 with Glut-1 in PDAC cells as demonstrated by multi-level experimental techniques, such as reciprocal co-immunoprecipitation (co-IP), proximity ligation assay (PLA), co-capping, and co-localization assays (Figs. 4a–f and 5). Results from reciprocal co-IP showed that MUC13 and Glut-1 remains in a molecular complex (Fig. 4a, b). This interaction stabilizes Glut-1 as demonstrated by cycloheximide chase assay with half-life as low as 1 h in MUC13-null P-V cells and greater than 3 h in MUC13-expressing P-M13 cells (Fig. 4c, d). PLA demonstrated that the interaction between these proteins is direct as both proteins exist in a close proximity as indicated by multiple red spots on the cell surface (Fig. 4e). MUC13 with HER2 serve as an experimental positive control in PLA as they are reported to physically interact in PDAC cells [12]. In addition, co-capping assay showed a progressively increasing distribution of both MUC13 and Glut-1 staining into membrane caps, which strongly suggests their co-clustering (yellow color) and molecular interaction in PDAC (Fig. 4f). Co-capping experiment, performed with α-tubulin in HPAF-II cells, did not show any clustering with MUC13, and served as negative experimental control. We further confirmed our observations through confocal immunofluorescence utilizing antibody-mediated double-immunofluorescence staining technique. Results of this analysis showed strong co-localization (yellow color) of MUC13 and Glut-1 in both HPAF-II and AsPC1 cells (Fig. 5a). Interestingly, this co-localization was abrogated upon treatment with a pharmacological inhibitor of NF-κB, sulphasalazine (Fig. 5b). Sulphasalazine is known to inhibit NF-κB activation/translocation to nucleus via direct inhibition of enzyme IκB kinase (IKK) that phosphorylates/inhibits NF-κB repressor, IκBα [17]. Similar results were obtained in reciprocal co-IP in which MUC13-expressing PDAC cells failed to form MUC13-Glut-1 molecular complexes in the presence of sulphasalazine (Fig. 5c, d). We further sought to investigate whether the interaction between the two proteins and NF-κB activation influence MUC13-mediated altered glucose metabolism in PDAC cells. We observed reduced lactate production in MUC13-expressing cells on treatment with sulphazalazine (Fig. 5e). These results imply that molecular interaction between MUC13 and Glut-1 is mediated through NF-κB activation and suggest the role of MUC13 in glucose metabolism via alteration of Glut-1 functionality in PDAC cells.

### MUC13 and Glut-1 expression directly correlate in pancreatic cancer tissues

**Fig. 6:**
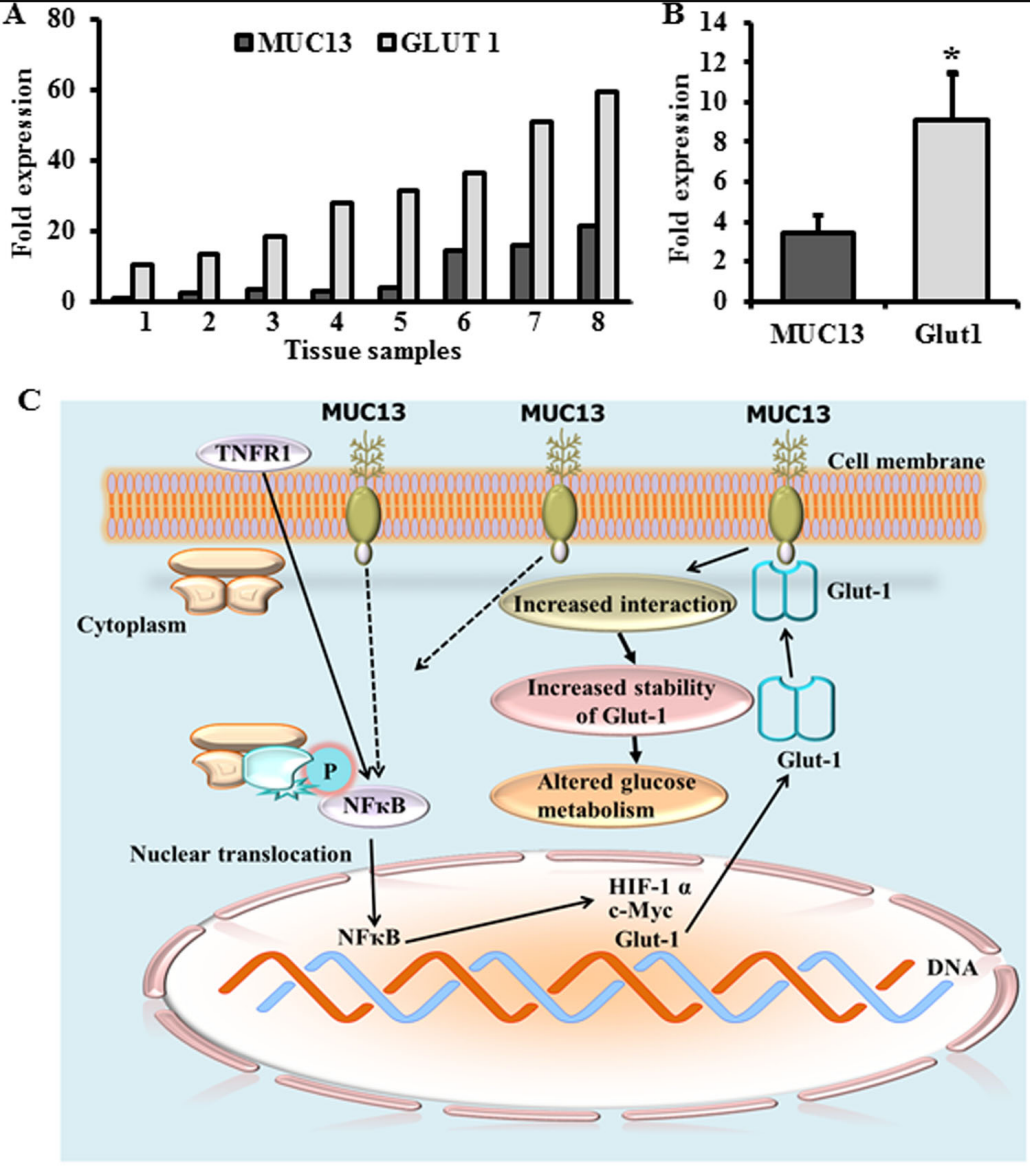
MUC13 and Glut-1 expression directly correlate in human pancreatic cancer tissues. **a** qPCR was performed to analyze the levels of MUC13 and Glut-1 using RNA from freshly collected human PDAC tissues (*N* = 8) procured from Baptist Memorial hospital, Memphis. **b** and cDNA from PDAC tissues (*N* = 6) procured from Origene. The correlation coefficient (*r*) and coefficient of determinations (*r*^2^) were 0.982 and 0.862, respectively. The analysis was performed by graph pad prism software. **c** Schematic representation showing the overall mechanism of MUC13 in modulating glucose metabolic network and driving tumorigenic microenvironment

To understand clinical correlation of MUC13-Glut-1 molecular interaction, we examined human pancreatic cancer tissues for MUC13 and Glut-1 expression using qPCR technique (Fig. 6a, b). Two independent sets of samples were utilized for the experiment, which include freshly collected RNA from PDAC tissue samples (*N* = 8) procured from Baptist Memorial hospital, Memphis (Fig. 6a); and cDNA (*N* = 6) procured from Origene (Fig. 6b). Analysis of MUC13 and Glut-1 expression in both the sets revealed a positive correlation between the expressions of these genes in human pancreatic cancer tissues. All tissue samples were positive for MUC13 and Glut-1, which showed a similar trend in the expression levels. The correlation coefficient (*r*) and coefficient of determinations (*r*^2^) were 0.982 and 0.862, respectively. The analysis was done using graph pad prism software.

## Discussion

High cellular glucose metabolism has been recently recognized as one of the hallmarks of cancer. Pancreatic cancer cells especially are highly active metabolically and programmed for extensively higher consumption of bio-fuels to operate oncogenic machinery. Increased glucose consumption of pancreatic cancer cells to meet the pathological requirements is supported by Warburg effect. This rewired metabolism is acquired to support their rapid proliferation and metastasis across the body. Although, several oncogenes and tumor suppressors are known to facilitate the metabolic adaptation in pancreatic cancer [18], investigation of novel molecular targets and their mechanisms are highly essential to improve treatment outcomes of this highly dreadful disease. Herein, we have identified MUC13 as a novel molecular target responsible for increased glucose metabolism in pancreatic cancer cells. Our study provides the first evidence for the critical involvement of MUC13 in metabolic reprogramming of pancreatic cancer cells by activating the NF-κB survival pathway and molecular interaction with Glut-1 receptor.

Our results demonstrate molecular mechanisms that drive the glycolytic phenotype conferring growth advantage to PanCa cells. This signifies a critical role of MUC13 in tumor microenvironment favoring the development of a more aggressive and invasive phenotype. Our study presents three important conclusions implicating the mechanisms that stimulate increased glucose metabolism and lactate production in pancreatic tumors. Our data first demonstrates that MUC13 expression alters the metabolic phenotype of PanCa cells and influences their proliferation and invasiveness due to enhanced lactate secretion. This as a result, provides an acidic tumor microenvironment, that contributes to highly aggressive and metastatic behavior of cancer cells [4] via influencing a number of oncogenic signaling pathways, including the activation of proteins that regulate glucose transport [19]. One such main protein is Glut-1, which represents a potential therapeutic target and its expression level is altered with MUC13 expression. 2-DG, a metabolic inhibitor of Glut-1 receptors, inhibits up to 59–95% growth of PDAC cells [20]. HIF-1α levels in MUC13-expressing cells increase under normoxic and hypoxic environments accompanied by the increase in lactate production. These results suggest that MUC13-induced altered glucose metabolism involves the activation of important proteins, such as Glut-1 and HIF-1α, which may be contributing to MUC13-mediated adaptive cellular mechanisms in tumor microenvironment to maintain a glycolytic phenotype. Second, our investigations reveal MUC13-induced activation of NF-κB survival pathway, which triggers a number of key proteins involved in altered glucose metabolism. Enhanced IκBα degradation and activation/nuclear translocation of NF-κB in MUC13-expressing cells was observed following TNF-α or OA stimulation. These events were typically accompanied by the activation of c-Myc, HIF-1α, and Glut-1 expression levels that are observed to be abrogated specifically in MUC13 cells following silencing of NF-κB (Fig. 6c). Our data indicate that the metabolic events controlled by MUC13 occur following NF-κB activation.

MUC13 has been recently reported to functionally interact with HER2 and this interaction mediates MUC13-induced pancreatic cancer progression. This study elucidated a novel, MUC13-Glut-1 interaction that may be one of the important events in driving mechanisms of aberrant glucose metabolism in PanCa cells. We found that the interaction between MUC13 and Glut-1 is governed by constitutive NF-κB activation. MUC13 interaction stabilizes Glut-1 as suggested by increased half-life of Glut-1 in MUC13-expressing cells compared to MUC13-null cells. Interaction between these two proteins appears to be direct and in very close proximity, as evident by co-IP and PLA assays, respectively. Since MUC13 and Glut-1 complex is dependent on nuclear translocation of NF-κB as it is prevented by Sulphasalazine treatment, suggest an opportunity to develop novel therapeutic strategies to attenuate aggressive/metastatic behavior of PanCa cells. However, it also leads to questions, as to how NF-κB activation promotes interaction between MUC13 and Glut-1, and which specific domains of both proteins are involved in this molecular interaction. Although, further investigations are warranted, we speculate that a cytoplasmic domain of MUC13 binds to Glut-1, as cytoplasmic domain of MUC13 is suggested to have role in cell signaling [21]. A positive correlation between the expression of MUC13 and Glut-1 in human pancreatic cancer tissues suggest clinical correlation of our proposed novel mechanism related to aberrant glucose metabolism in pancreatic tumors.

In summary, this study revealed a novel role of MUC13 in rewiring of a distinct glucose metabolic network that drives favorable tumor microenvironment and oncogenic signaling pathways in pancreatic cancer cells for the adoption of their enhanced tumorigenic and metastatic behavior. Thus, inhibition of MUC13 via conventional pharmacological agents and/or non-conventional small interfering RNA/microRNA-mediated technologies can selectively suppress pancreatic tumor growth and metastasis via inhibition of glycolytic influx in PanCa cells. Our observations suggest that the reliance of pancreatic tumor growth and metastasis on MUC13-Glut-1 mediated glucose metabolism can be exploited for the development of novel targeted therapeutic strategies for pancreatic cancer. Finally, our study offers a very promising target for therapeutic manipulation via disabling aberrant glucose metabolism in pancreatic cancer patients, thereby prolonging survival rate of patients suffering from this dreadful disease.

## Materials and methods

### Cell culture and generation of MUC13-expressing stable cell lines

MUC13-null pancreatic cancer cells (P-V, M-V), and stably expressing MUC13 (P-M13, M-M13) were used for the study, as described earlier [11]. We also generated MUC13-expressing HPAF-II (sh-V) and knockdown (sh-M13) cells using a lentiviral transfection, for our study. Cells were maintained in DMEM or DMEM/F12 media supplemented with 10% fetal bovine serum and 100 µg/ml of G418 antibiotic or 5 µg/ml puromycin. These cells were grown at 37 °C in an incubator which supplied 5% CO_2_ in a humidified environment.

### Clinical samples

Human pancreatic cancer tissue samples were procured from Baptist Memorial Health Center. In addition, cDNA of pancreatic cancer tissue specimens was purchased from Origene.

### Immunoblotting

Whole-cell lysates were prepared followed by immunoblotting as described earlier [12,22,23,]. The expression level of several proteins were analyzed using specific primary antibodies obtained from Cell Signaling: NF-κB p65 (catalog number: 8242), phospho-NF-κB p65, Ser^536^ (catalog number: 3033), IκBα (catalog number: 4814), phospho-IκBα, Ser^32/36^ (catalog number: 8219), Histone H3 (catalog number: 4499), GAPDH (catalog number: 5174), HIF-1 α (catalog number: 3716), Bcl-2 (catalog number: 4223), c-Myc (catalog number: 9402), Glut-1 (catalog number: 12939), alpha-tubulin (catalog number: 2144), p27 Kip1 (catalog number: 2552), Ras (catalog number: 3965), and KRAS-12D (catalog number:14429). The anti-MUC13 monoclonal antibody used for this manuscript was produced in our lab. The secondary antibodies for rabbit (catalog number: 4011), and mouse (catalog number: 4021) conjugated with horseradish peroxidase were obtained from Promega.

### Reciprocal co-IP

The reciprocal co-IP experiments were performed using AsPC1 and HPAF-II cells, which express MUC13. 500 µg of protein was used and incubated with antibodies at 4 °C overnight. Immunoprecipitates were eluted with SDS sample buffer obtained from Santa Cruz Biotechnology, TX, USA following the procedures as described earlier [12,24,].

### Confocal immunofluorescence

Confocal immunofluorescence was performed as described earlier [12,23,]. Briefly, cells were fixed and incubated with respective primary antibodies overnight. This was followed by incubation with Cy3, or Alexa Fluor 488, donkey secondary antibodies for 1 h. The images were then captured at ×400 magnification using a confocal microscope (Nikon Corporation, Melville, NY, USA).

### Wound-healing assay/scratch assay

Cell migration assay was performed using wound-healing assay [22,25,]. Cells were seeded in 12-well plate at a density of 2 × 10^5^ cells. The cells were treated with l-lactate (2 mM) and 2-DG (10 mM) and further incubated for 24 h. The images were captured at 0 and 24 h.

### Invasion assay

Cell invasion assay was performed using BD Biocoat Matrigel Invasion Chambers (BD Biosciences), as described earlier [22]. Cells were seeded in matrigel invasion chambers in a serum free medium. Cells were treated with l-lactate (2 mM) and 2-DG (10 mM) followed by incubation for 24 h. Then, cells were fixed using methanol and were stained with crystal violet. The images were captured at 24 h.

### Cell proliferation assay

Cell proliferation assay was performed in MUC13-null (P-V) and MUC13-expressing (P-M13) cells after treatment with lactate (2 mM) and 2-DG (10 mM). The experiment was terminated after 48 h with the addition of 20 µl of tetrazolium dye, MTT reagent: 3-(4,5-dimethylthiazol-2-yl)-2,5-diphenyltetrazol-2-yl)-2,5-di-phenyltetrazolium bromide. After 2 h, 100 µl of dimethylsulfoxide was added to the cells and was kept on shaker for 10 minutes. Finally, the absorbance was recorded at 570 nm, and the percentage cell viability was quantified.

### Lactate and glucose assays

Lactate and glucose assays were performed using kits from Cayman Chemicals (Lactate assay kit catalog number: 600450, Glucose assay kit catalog number: 10009582). MUC13-expressing and MUC13-null cells were seeded (10^4^ cells/well in 96-well plate) and media collected to measure the amount of lactate after 24 h, and unused glucose levels after 48 h. The samples were analyzed according to the instructions provided in the kit, the readings were recorded and calculations were done.

### Isolation of RNA and PCR

RNA from pancreatic cancer cells and tissues was isolated using Qiagen kit and quantified using Nanodrop instrument 2000 (Thermo Scientific). RNA (2 µg) was reverse transcribed using SYBR green RNA Reverse Transcription kit. The expression of genes was determined by using specific primers by qPCR and semi-quantitative PCR. GAPDH was used as an internal control for the reactions.

### In situ PLA

This assay was performed using the Duolink Red Starter PLA Kit as described earlier [12] to detect the subcellular localization of the protein-protein interaction at a single-molecule resolution. Briefly, oligonucleotide-conjugated anti-mouse minus and anti-rabbit plus PLA secondary probes were added and incubated in a humidified chamber for 1 h at 37 °C. The oligonucleotides were ligated for 30 min at 37 °C to produce rolling circle amplification products tagged with a red fluorescence probe. Furthermore, the nuclei were counterstained with 4’, 6-diamidino-2-phenylindole, and were then visualized using a Zeiss confocal microscope.

### Co-capping assay

This assay was performed using MUC13-expressing HPAF-II and AsPC1 cells as described before [12]. The cells were incubated with NHS-Rhodamine-labeled anti-MUC13 mAb (30 µg/ml) for 1 h at 4 °C, and plated for adherence at 37 °C for about 2 h. The cells were incubated with Glut-1 (Cell Signaling, catalog number: 12939), or α-tubulin negative control (Cell Signaling, catalog number: 2144), overnight. Cells were further incubated with Alexa Fluor 488 anti-Rabbit secondary antibody, and the images captured using 710 Zeiss confocal microscope [23].

### Statistical analysis

The data in this manuscript were evaluated and analyzed using Microsoft excel software, and the statistical analysis is represented as means + SEM of three independent experiments. In all cases, the significance of the data was analyzed through unpaired two-sided Student’s *t*-test, and *p*-value <0.05 was considered statistically significant.

## Electronic supplementary material

Supplementary data

Supplementary Figure 1

Supplementary Figure 2

Supplementary Figure 3

Supplementary Figure 4

Supplementary Figure 5
