# Supplementary material for: MUC13 contributes to rewiring of glucose metabolism in pancreatic cancer
Source: Oncogenesis. 2018 Feb 22;7(2):19. doi: 10.1038/s41389-018-0031-0 (PMC5833644; doi:10.1038/s41389-018-0031-0)
Supplement: Supplementary file 1 — Supplementary data [file 41389_2018_31_MOESM1_ESM.docx]

**Supplementary Figure S1.** MUC13 inhibits glucose metabolism in cells. (A) Western blotting depicting expression of MUC13 in MUC13 expressing (sh-V) and knockdown (sh-M13) cells. (B) Lactate and (C) Glucose assay in the indicated cells. (D) Cell proliferation assay in MUC13 null (P-V) and MUC13 expressing (P-M13) cells after treatment with lactate (2 mM) and 2-DG (10 mM). Assay was terminated after 48 hrs and the percentage cell viability was quantified. *p<0.05.

**Supplementary Figure S2.** MUC13 enhances expression of c-Myc and Glut-1 in cells. Confocal microscopy images showing the expression of (A) c-Myc and (B) Glut-1 in indicated cells. Cells were seeded in 4-well chambered slides, fixed, permeabilized and stained with respective antibodies. The slides were further processed and analyzed for confocal microscopy. Images were captured at 400X. DAPI was used as a counter stain for the nucleus.

**Supplementary Figure S3.** MUC13 enhances expression of HIF-1 α in cells. Confocal microscopic images showing the expression of HIF-1 α in indicated cells. Cells were seeded in 4-well chambered slides, fixed, permeabilized and stained with HIF-1 α. The slides were analyzed for confocal microscopy. DAPI was used as a counter stain for the nucleus.

**Supplementary Figure S4.** Mean fluorescence intensities of the confocal images for (A) c-Myc, Glut-1 (from Figure S2) and (B) HIF-1 α expression (from Figure S3). Zen software was used for quantifying the fluorescence intensity of the images. *p<0.05.

**Supplementary Figure S5.** MUC13 expression leads to the OA-induced activation/nuclear translocation of NFҡB p-65. (A) Western blots showing the expression level of IκB α in cytoplasmic lysates from MiaPaca (M-V/M-M13) cells. Cells were stimulated with Okadaic acid at indicated concentrations (0, 50 and 100 nM) for 4 hours. (B) Western blots showing the expression level of p65 and phospho-p65 in the nuclear lysates of aforementioned cells. Histone H3 served as the internal control.
