# Supplementary figures and images for: MUC13 contributes to rewiring of glucose metabolism in pancreatic cancer
Source: Oncogenesis. 2018 Feb 22;7(2):19. doi: 10.1038/s41389-018-0031-0 (PMC5833644; doi:10.1038/s41389-018-0031-0)

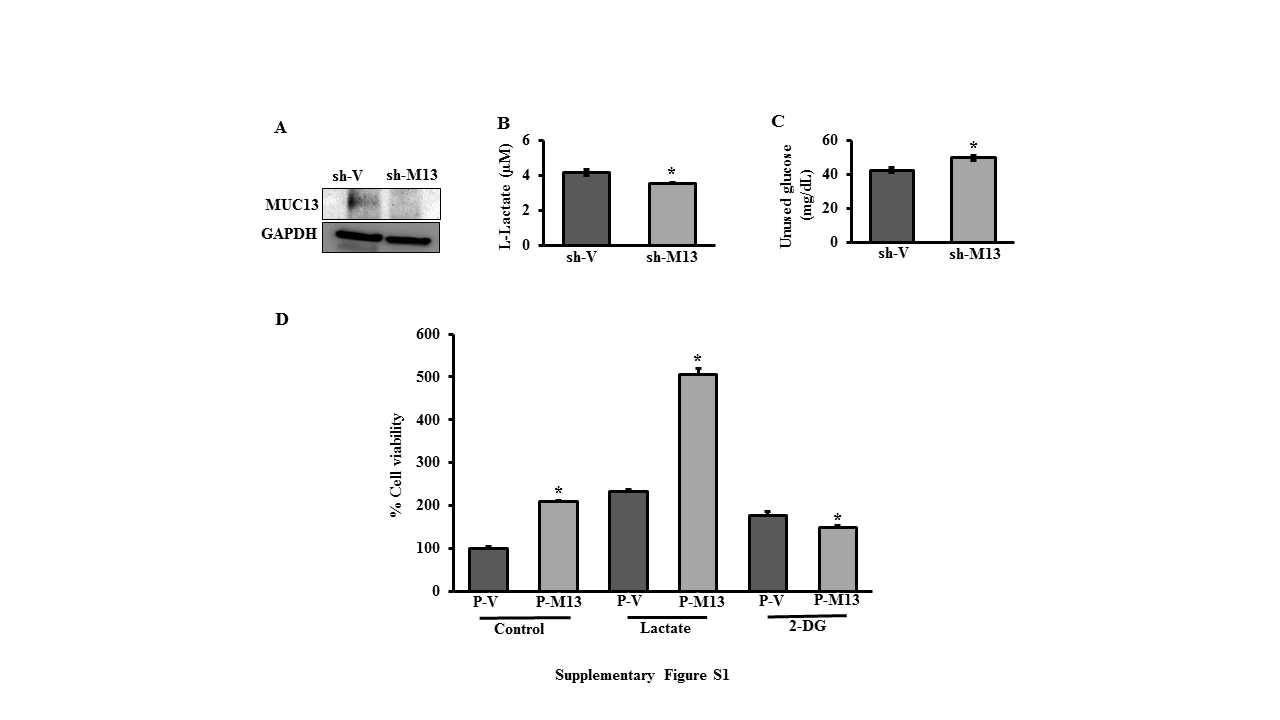

Supplement: Supplementary file 2 — Supplementary Figure 1 [file 41389_2018_31_MOESM2_ESM.tif]

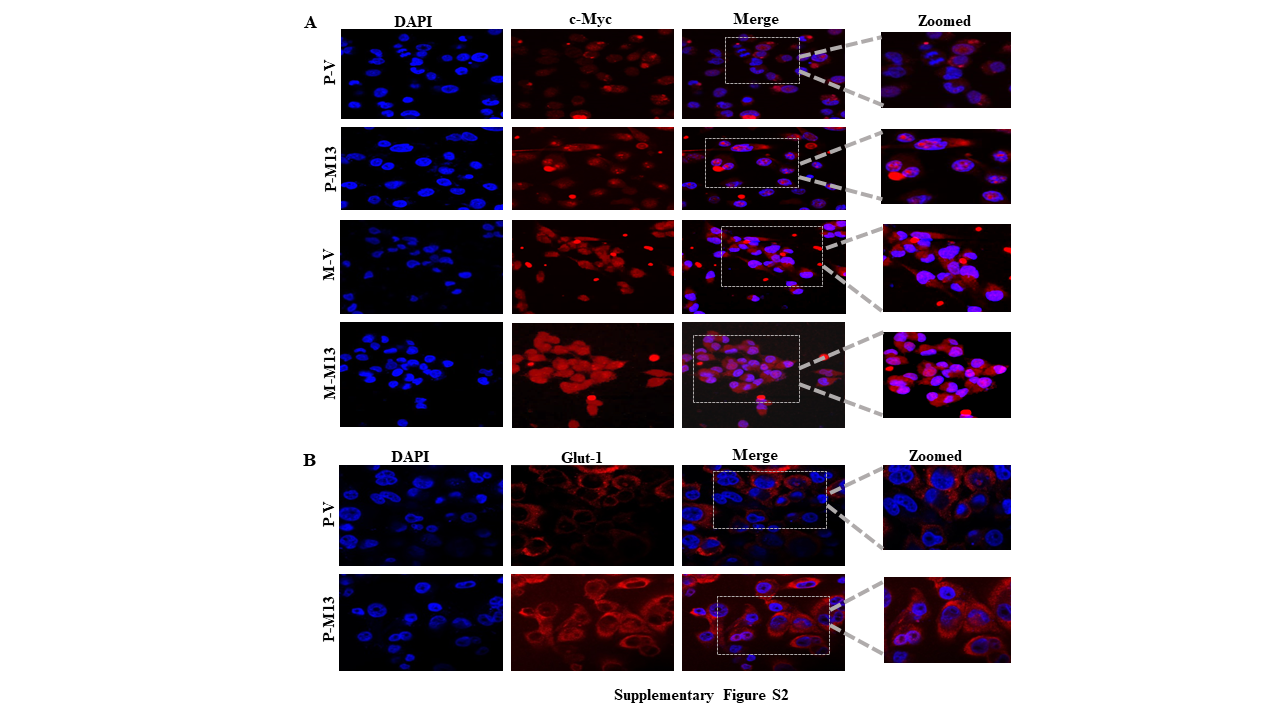

Supplement: Supplementary file 3 — Supplementary Figure 2 [file 41389_2018_31_MOESM3_ESM.tif]

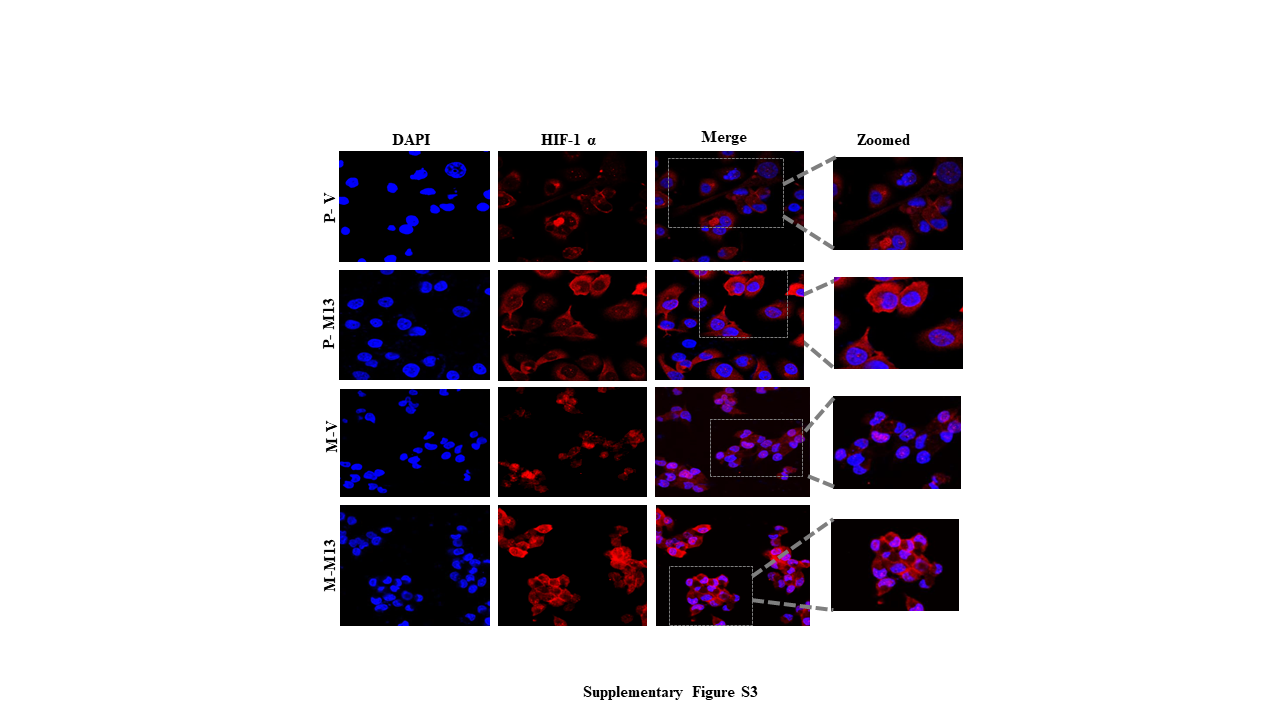

Supplement: Supplementary file 4 — Supplementary Figure 3 [file 41389_2018_31_MOESM4_ESM.tif]

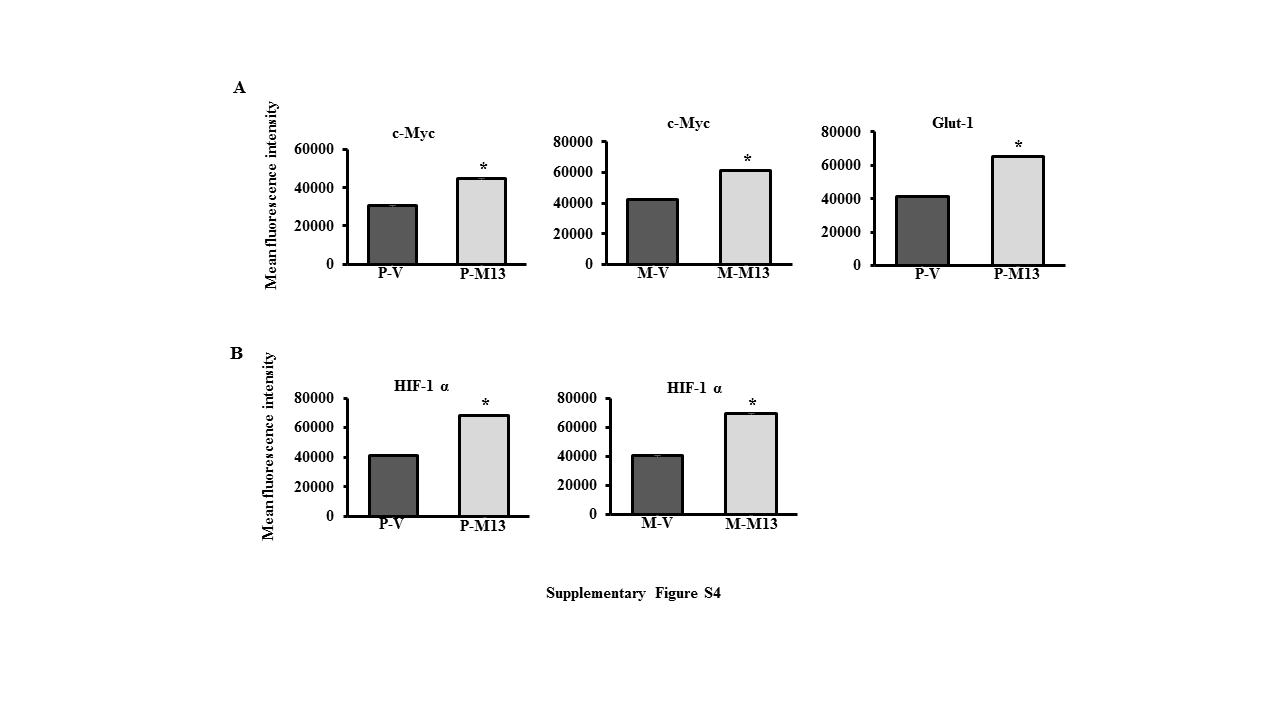

Supplement: Supplementary file 5 — Supplementary Figure 4 [file 41389_2018_31_MOESM5_ESM.tif]

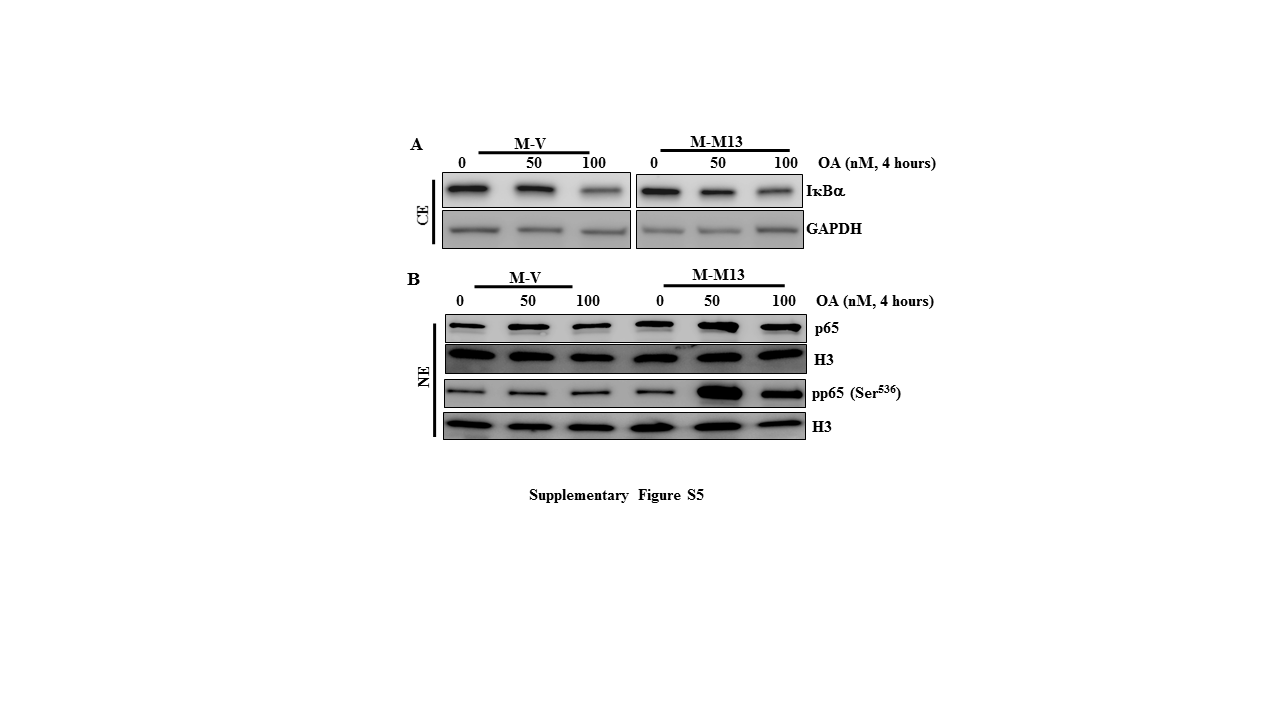

Supplement: Supplementary file 6 — Supplementary Figure 5 [file 41389_2018_31_MOESM6_ESM.tif]
